# Household Factors Associated with Self-Harm in Johannesburg, South African Urban-Poor Households

**DOI:** 10.1371/journal.pone.0146239

**Published:** 2016-01-05

**Authors:** Nisha Naicker, Pieter de Jager, Shan Naidoo, Angela Mathee

**Affiliations:** 1 Environment & Health Research Unit, South African Medical Research Council, Johannesburg, South Africa; 2 Department of Community Medicine, School of Public Health, University of the Witwatersrand, Johannesburg, South Africa; 3 Epidemiology and Surveillance Unit, National Institute for Occupational Health, National Health Laboratory Service, Johannesburg, South Africa; 4 Faculty of Health Sciences, University of Johannesburg, Johannesburg, South Africa; The George Institute for Global Health, INDIA

## Abstract

**Introduction:**

Low and middle income countries bear the majority burden of self-harm, yet there is a paucity of evidence detailing risk-factors for self-harm in these populations. This study aims to identify environmental, socio-economic and demographic household-level risk factors for self-harm in five impoverished urban communities in Johannesburg, South Africa.

**Methods:**

Annual serial cross-sectional surveys were undertaken in five impoverished urban communities in Johannesburg for the Health, Environment and Development (HEAD) study. Logistic regression analysis using the HEAD study data (2006–2011) was conducted to identify household-level risk factors associated with self-harm (defined as a self-reported case of a fatal or non-fatal suicide attempt) within the household during the preceding year. Stepwise multivariate logistic regression analysis was employed to identify factors associated with self-harm.

**Results:**

A total of 2 795 household interviews were conducted from 2006 to 2011. There was no significant trend in self-harm over time. Results from the final model showed that self-harm was significantly associated with households exposed to a violent crime during the past year (Adjusted Odds Ratio (AOR) 5.72; 95% CI 1.64–19.97); that have a member suffering from a chronic medical condition (AOR 8.95; 95% 2.39–33.56) and households exposed to indoor smoking (AOR 4.39; CI 95% 1.14–16.47).

**Conclusion:**

This study provides evidence on household risk factors of self-harm in settings of urban poverty and has highlighted the potential for a more cost-effective approach to identifying those at risk of self-harm based on household level factors.

## Introduction

Globally, the burden of self-harm (defined in this paper as suicide or attempted suicide) has increased by 24% since the first global burden of disease estimates in 1990.[1] Of the 291 conditions assessed, self-harm was found to be the 18^th^ leading contributor globally and the 27^th^ leading contributor in southern Sub-Saharan Africa in terms of disease burden.[1] However, this is probably an underestimation of the true burden, especially in Africa, as very few Sub-Saharan countries produce any epidemiological data on self-harm.[2, 3]

Despite low and middle income countries (including upper middle income countries such as South Africa) accounting for the majority of the global burden of self-harm,[4–6] most knowledge on the subject is based on research from high income countries. A global review on the role of psychiatric diagnosis of suicide by Bertolote and colleagues[7] found that 82,2% of academic papers were from North America or Europe. Due to the traditional high-income country bias in the published academic literature, a number of well-recognized risk factors for self-harm have recently been shown to be dubious in lower or middle-income countries. For example, evidence from China[8] and India[9] has dispensed with the prevailing belief, based on evidence from some high income countries, that men are more likely to commit suicide, or that marriage is a protective factor against self-harm.[6] The 2014 WHO Report on Preventing Suicide stated that in high income countries the male to female ratio is 3.5 and in LMIC the ratio is 1.6. However in LMIC in Africa the ratio increases to 2.5, highlighting the differences between regions. Similarly, the rural suicide rates in these countries are higher than the urban rates whereas in high-income countries the urban rural difference varies over time.[5, 10–11] Thus evidence from lower and middle-income countries broadens our understanding of the complex socio-economic and cultural dimensions of self-harm. There has, however, been relatively little work done on this important public health problem and it is not perceived as a research priority in Africa. For example, a multi-country survey of research priorities for mental health in low and middle-income countries found that suicide was significantly under prioritized in terms of its burden.[12]

Violence and injury are the second leading cause of death and disability in South Africa, with suicide constituting an important share of this burden.[13] Suicide was ranked 11^th^ overall in the 2000 initial burden of disease estimates for South Africa.[14] Males in South Africa have a higher, and females a lower, suicide rate than the global average,[15] with an estimated overall suicide rate of between 11.5 and 25 per 100 000.[16] It has been estimated that up to 7 582 South Africans die each year from self-harm with up to 151 646 suicide attempts each year.[17]

As with research from high income countries, research from South Africa and Africa in general have focused individual-level risk factors for self-harm such as gender, age, marital status, employment, education levels, income status, poverty levels, chronic pain, mental disorders and family history of mental disorders or suicide. [10, 18]. Studies have also shown that factors such as experiencing discrimination or feeling isolated within a community, abuse or exposure to violence are also potential risk factors for self harm.[10] Several theories (social, psychological and biological) have tried to explain the risk for attempting suicide at an individual level. [19] Studies have shown that successful suicide prevention programs include screening in clinical settings [18]. However in a country like South Africa where mental health screening and treatment services are limited in primary health care clinics, the resources for identification of individual risk factors and interventions may not always be possible. Prevention campaigns can be aimed at a broader household and community level is achievable since it requires less human and financial resources. However research into the risk factors at household level is not available and thus cannot inform prevention strategies. This paper, aims to describe environmental, socioeconomic and demographic risk factors for self-harm at the household level in five urban communities in Johannesburg (South Africa), which may prove to be a more effective approach in the prevailing circumstances. The risk factors described in this study have been shown to be significant contributors to the self-harm in individuals.

## Methods

### Data collection

The Health, Environment and Development (HEAD) study is an annual cross-sectional study conducted in five settlements in Johannesburg, South Africa. The aim of the HEAD study is to monitor changes in living conditions and health status in five sentinel sites over a 10-year period from 2006 to 2015. In Braamfischerville, Riverlea, Hospital Hill and Bertrams dwellings were randomly selected in 2006 using a table of random numbers and town planning maps of the study areas. In Hillbrow, apartment buildings were first randomly selected, followed by floors in the selected buildings and then apartments on the selected floors. The first survey was conducted in 2006 with all subsequent surveys being conducted with persons residing in the pre-selected dwelling; therefore household inhabitants may change from year to year. The study population comprises respondents and other members of the selected primary households in the study sites. A household member was defined as someone who usually eats with the other members (shares meals). Table 1 provides a summary of the study site characteristics and response rates.

**Table 1:** Summary of study site characteristics.

Study Site	Brief description	Number of households selected	Average response rate (2006–2011)
Braamfisherville	post-apartheid, low-cost housing	188	67%
Riverlea	apartheid era, low-cost housing	158	65%
Hospital Hill	an informal settlement on the city periphery	188	58%
Bertrams	old inner-city suburb	132	42%
Hillbrow	densely populated, high-rise inner city area	142	51%

Data were collected through administration of a pre-structured questionnaire by trained environmental health students from the University of Johannesburg and included information on demography and socio-economic status; migration patterns; perceptions of housing and neighborhood conditions; hygiene behavior; quality of life; exposure to violence; physical activity and health status (acute; chronic and mental). Only one member of each household was interviewed each year. Ethical approval for this study was obtained from the University of the Witwatersrand Human Research Ethics Committee (Medical). Ethics approval number: M10471. Questionnaires are administered only after obtaining written, informed consent from respondents. Written informed consent is a requirement for approval from the Ethics committee and the South African Medical Research Council.

Dependent and independent variables: Fatal and non-fatal suicide attempts were self-reported with respondents in each of the households being asked: “In the past 12 months has anyone in the household attempted suicide and survived/did not survive”. The dependent variable, self-harm, was defined as cases of fatal (n = 31) and non-fatal suicide (n = 55) attempts. There was no difference in risk factors between cases of fatal and non-fatal attempts thus both categories were combined to form a single variable. Independent variables included community characteristics (study site) and household level characteristics (socio-economic; environmental; health status and crime). Household-level variables were the primary independent variables of interest. These variables were chosen because at an individual level they have been shown to be risk factors. However as individual level data was not available, household level data was adapted for use in the analyses. Data such as health status, experiences of crime, socio-economic profiles and environmental conditions were self-reported by a member of the household. For example participants were asked “In the past 12 months, has anyone in the household experienced/been a victim of the following crime in your neighbourhood?*”* A list of violent crimes (rape, gunshot injury, stabbing, robbery with aggravating circumstances) and non-violent crimes (theft, burglary, hi-jacking) was provided. In order to assess dwelling quality, a composite measure was developed from sixteen questions which including questions on dampness and leaking roofs; overcrowding; odours; cracked walls and peeling paint. Each question on dwelling quality was given an equal rating. Households that reported below average (below the sample mean score) quality dwellings were classified as “poor” quality dwellings. Dwelling quality and access to services e.g. water and sanitation were used as indicators of poverty.

### Data analysis

HEAD study data from 2006–2011 were combined to describe the prevalence of suicide over 6 years in each of the study areas and to assess household-level risk factors for self-harm. Data were analyzed in STATA version 12 (StataCorp 2011). In order to minimize the effect of clustering and adjust for the study design, the data for each site was weighted and the STATA survey command used for analysis. The trend analysis however was not conducted using the STATA survey command. After the datasets were combined and weighted for study site and response rates, frequencies of various exposures were calculated in those households who had reported a case of self-harm during the preceding twelve months and those who did not. Bivariate analysis was conducted to determine which factors were significantly associated with self-harm. Significance was taken at p < 0.05. In order to find the most parsimonious set of risk factors and account for confounding factors, forward stepwise multiple logistic regression was undertaken considering all independent variables which showed significance at p < 0.25 during bivariate analysis. The final model included risk factors that were significant at p < 0.05.

## Results

Over the period 2006–2011 a total of 2 795 households were interviewed. Eighty six (86) cases of self-harm were reported over the six-year period of which 31 cases had a fatal outcome. The total 6-year household prevalence of self-harm was 3.1% (N = 86/2792) with no significant change over the six-year period (*nptrend*; *p* = 0.612).

With reference to Table 2, respondents from Riverlea, Bertrams, Braamfischerville and Hospital Hill were significantly more likely to report a case of self-harm compared to Hillbrow. In assessing household-level demographic and socio-economic risk factors, it was found that households with more than four members were more likely to report self-harm. Although an increase in the number of children under the age of five years in a household was associated with an increased likelihood of self-harm, this was not a significant association. Employment status, educational status, membership of a faith-based organization and food insecurity was not associated with self-harm. A non-significant decrease in self-harm was found with rising household income and a non-significant increase in self-harm was associated with receipt of a social grant. Households in which the primary decision maker was female had a significantly higher chance of a reported case of self-harm during the preceding year compared to households with male decision makers.

**Table 2:** Frequencies and bivariate analysis of household demographic, socio-economic and environmental factors potentially associated with self-harm (2006–2011).

Variable	Prevalence of reported self-harm *n (%) N = 86/2792*	*p*-value	Confidence interval
Study site			
Hillbrow	2/431 (0.46)		
Hospital Hill	14/658 (2.13)	0.044	0.133–3.027
Braamfischerville	25/758 (3.30)	**0.009**	0.429–3.423
Bertrams	12/333 (3.60)	**0.007**	0.534–3.568
Riverlea	33/612 (5.39)	**0.001**	1.012–3.922
Number of household members			
2 or less	10/611 (1.64)		
3–4	27/1,142 (2.36)	0.223	0.734–3.774
more than 4	49/1,035 (4.74)	**0.005**	1.432–7.262
Number of children under 5 years			
None	34/1,314 (2.59)		
One	22/628 (3.50)	0.328	0.748–2.380
Two or more	14/280 (5.00)	0.27	0.712–3.353
Household member has tertiary education			
None	74/2,415 (3.06)		
At least one member	12/377 (3.18)	0.928	0.507–2.105
At least one household member has full-time employment			
No	35/1,183 (2.96)		
Yes	51/1,609 (3.17)	0.625	0.684–1.879
Household Income			
<1000	34/867 (3.92)		
1001–5000	38/1,186 (3.20)	0.866	0.414–2.103
>5000	9/335 (2.69)	0.723	0.520–2.562
Government grant*			
None	18/415 (4.34)		
At least one grant	8/147 (5.44)	0.241	0.676–4.736
Food Security			
Food Secure	9/481 (1.87)		
Food Insecure	77/2,311 (3.33)	0.224	0.747–3.469
Gender of primary household decision maker			
Male	25/1,147 (2.18)		
Female	49/1,178 (4.16)	**0.004**	1.307–3.923
Joint	12/443 (2.71)	0.624	0.56–2.552
Belonging to religious group			
Yes	63/1,883 (3.35)		
No	16/745 (2.15)	0.142	0.328–1.174
Dwelling type			
Formal	70/2,186 (3.20)		
Informal	13/508 (2.56)	0.288	0.371–1.342
Quality of dwelling			
Good	45/1,721 (2.61)		
Poor	20/337 (5.93)	**0.005**	1.292–4.316
Water			
In house tap	69/2,035 (3.39)		
Other	17/749 (2.28)	0.294	0.381–1.339
Electricity used for cooking			
Yes	74/2,209 (3.24)		
No	12/498 (2.41)	0.627	0.419–1.689
Sanitation			
Flush toilet	72/2,109 (3.41)		
Other	14/666 (2.10)	0.252	0.354–1.313
Smoking inside the house			
No	38/1,550 (2.45)		
Yes	36/699 (5.15)	**0.01**	1.182–3.337

* For households with incomes of less than R1000 per month

In terms of crime (Table 3) it was found that households in which a member had been the victim of a violent crime (Crude OR 10.94; 95% CI 6.60–18.13); victim of non-violent crime (Crude OR 5.77; 95% CI 3.60–9.25) and households with a member convicted of a crime (Crude OR 4.35; 95% CI 2.25–8.40) were signifcantly more likely to report self-harm.

**Table 3:** Frequencies and bivariate analysis of violence and health-status as factors potentially associated with self-harm (2006–2011).

Variable	Prevalence of reported self-harm	*p*-value	Confidence interval
	*n (%) N = 86/2792*		
Household member victim of violent crime (in last year)			
No	25/2,164 (1.16)	** **	
Yes	61/628 (9.71)	**<0.001**	6.597–18.127
Household member victim of non-violent crime (in last year)			
No	34/2,162 (1.57)		
Yes	52/630 (8.25)	**<0.001**	3.597–9.250
Household member convicted of crime*			
No reported conviction	53/1,996 (2.66)		
Reported conviction	18/201 (8.96)	**<0.001**	2.254–8.395
Household member suffers from chronic disease^§^			
No	63/2,500 (2.52)		
Yes	23/292 (7.88)	**<0.001**	2.147–6.445
Household member suffers from mental illness			
No	60/2089(2.87)		
Yes	9/79 (11.39)	**<0.001**	2.609–11.821
Death in household in last year^#^			
No	39/2,433 (1.58)		
Yes	14/223 (6.28)	**<0.001**	2.041–9.990

* This question was not included in the 2006 data collection tool

^§^ Cancer/ Cerebrovascular Accident (stroke)/ HIV/AIDS/ Disability

^#^ Excludes death due to suicide

Households reporting a member suffering from a chronic medical condition (cancer; stroke; disability or HIV/AIDS) (Crude OR 3.72; 95% CI 2.15–6.45) or a psychiatric illness (Crude OR 5.55; 95% CI 2.61–11.82) were significantly more likely to report a case of self-harm than households without. Furthermore, a death in the household during the preceding year (excluding death due to suicide) was associated with self-harm (OR 4.52; 95% CI 2.04–9.99).

Household-level environmental factors potentially associated with self-harm are presented in Table 2. Although the type of dwelling was not associated with an increased likelihood of self-harm, the quality of the dwelling was. Those who perceived the quality of their dwelling to be poor had an increased likelihood of reporting an incident of self-harm (OR 2.36; 95% CI 1.29–4.32). Access to basic amenities (water, electricity and sanitation) showed no association with self-harm.

Findings from the stepwise multiple logistic regression model are presented in Table 4. The final model included the independent variables “victim of a violent crime”, “household member suffering from a chronic medical condition” and “smoking inside house”. Households with a member who had been the victim of a violent crime during the preceding year were more than five times (AOR 5.72; 95% CI 1.64–19.97) more likely to report a case of self-harm. Similarly, households with a member suffering from a chronic medical condition had a significantly higher likelihood of reporting an incident of self-harm (AOR 8.95; 95% CI 2.39–33.56). Respondents who reported that one or more household members smoked inside the house were more than four times (AOR 4.39; 95% CI 1.14–16.47) more likely to report an incident of self-harm.

**Table 4:** Factors associated with self-harm by stepwise multiple logistic regression: Johannesburg, South Africa (2006–2011).

Risk Factor	Adjusted OR	*p*-value
	(95% CI)^#^	
Household member victim of violent crime (in last year)		
No	1	
Yes	5.72 (1.64–19.97)	**0.006**
Household member suffers from chronic disease*		
No	1	
Yes	8.95 (2.39–33.56)	**0.001**
Smoking inside house		
No	1	
Yes	4.39 (1.14–16.47)	**0.031**

^**#**^ Adjusted for study area and model covariates

* Cancer/ Cerebrovascular Accident (stroke)/ HIV/AIDS/ Disability

## Discussion

This paper describes the prevalence of self-harm rates for five socio-economically vulnerable urban communities in Johannesburg, and identifies household-level risk factors for self-harm. It has shown that household-level risk factors are potentially important predictors of self-harm.

In this study household exposure to violence has been highlighted as strongly associated with self-harm. Stressful life events, such as exposure to violence, are recognized as proximal risk factors for self-harm in both high and low to middle income countries.[20, 21] While this study could not discern between intra-household and broader community violence, intimate partner violence has been shown to be strongly associated with self-harm in women[22–24] and widely prevalent in South Africa where almost 20% of male perpetrators of intimate partner homicide died by suicide within one week of the crime.[25]

Although, at a household level and after adjusting for confounding factors, psychiatric illness of a household member was not found to be a risk factor of self-harm in this study, chronic illness was. Psychiatric health and chronic physical illness such cardiovascular and chronic respiratory diseases as well as neurological conditions have been established individual-level risk factors for self-harm in international studies,[26–28]. In South Africa, HIV has been associated with an increased rate of suicide attempts, however the relationship between other chronic illnesses and self-harm has not been extensively researched in South Africa.[29] Our study provides evidence in support of international findings, and suggests that experiencing violence and chronic disease not only increases the risk of self-harm in the affected individual, but could also increase risk in other household members. The chronic disease burden in South Africa is increasing [30] and thus this risk factor for self harm could negatively impact suicide rates.

Housing is an important social determinant of health and has been shown to impact on psychiatric health and self-harm.[31] We found indoor smoking to be associated with self-harm. Previous studies have found smoking and exposure to second hand smoke to be an independent predictor of self-harm among adolescent and young adults.[32–36] Per capita tobacco consumption has also been positively associated with suicide rates.[37] Smoking may act as a coping mechanism for people undergoing stressful life events or those with underlying psychopathology,[38] thus partially explaining the association between tobacco use and self-harm. Research has also shown that smoking increases the risk of chronic debilitating conditions that in turn increases the risk for self harm. Smoking and exposure to second hand smoke has been shown to decrease serotonin levels which is associated with depression. [36]

Evidence from higher income countries shows smaller households, and especially single-member households, to be at increased risk for self-harm.[39–41] By contrast, we found that an increase in household size was positively associated with an increased risk of self-harm. Material demands placed on scarce household resources by additional household members may act as a psychosocial stressor. A study from Ghana has also demonstrated a positive correlation between household size and psychological disorders.[42] However it must be noted that as household size increases the more likely a rare event such as suicide has a potential to occur because of the increase in numbers of the population under study.

Unemployment, lower levels of education and socio-economic status have all been associated with self-harm.[43–46] Our study found no significant relationship between level of education or employment and self-harm, but the analysis suggested that as household incomes rise—for these very poor households—self-harm risk decreases. Food security also seemed to incur protection against self-harm: a finding in line with evidence from India.[47] The prevalence of socio-economic vulnerability in each of the five study sites is extremely high and may partially explain the lack of significant associations between socio-economic vulnerabilities and self-harm.

Although religious affiliation has been reported to have an individual-level protective effect against self-harm in low and middle income countries, including South Africa,[48] no such effect was found at a household-level in our study. This may be due to the fact that the religious affiliation was reported on a household level and thus variations in individual household members’ degrees of religious affiliation was lost: most households (72%) reported some religious affiliation.

This study had a number of limitations. Our outcome measure of interest was drawn from one question on self-harm and has not been validated. We relied on retrospective self-reported cases of self-harm and therefore our findings are susceptible to recall bias and misclassification. Cases of self-mutilation, for example, may have been misinterpreted by household respondents as suicidal behavior. Household respondents may have under-reported incidents of self-harm, because they were unaware of the incident or reluctant to discuss a painful family or household event. Findings may also have been affected by incomplete data and lower response rates in some study sites (Bertrams and Hillbrow), which were affected during 2008/2009 when a wave of xenophobic violence plagued South Africa.[49] Further, the cross-sectional study design limits our conclusions on the causality between various explanatory variables and self-harm. No data were available on individual household members who engaged in self-harm, therefore we are not able to comment on any individual risk factors for self-harm or the means by which individuals attempt or die by suicide in these communities.

Despite the limitations, the study has highlighted the impact of household risk factors on self-harm and provides an evidence base for suicide risk prevention measures aimed at the household and community. This involves a multidisciplinary public health approach that involves crime prevention, awareness campaigns regarding mental well-being and prevention of chronic lifestyle related diseases.

## Conclusion

In this study household risk factors (chronic diseases, indoor smoking and exposure to violence) for self-harm in settings of poverty in Johannesburg were examined. Currently screening for elevated risk of self-harm is done mainly at the individual level, which is relatively costly. While further research is necessary, this study points to the potential for a more cost-effective approach to identifying those at risk of self-harm based on household level factors, such as a chronic ill health condition or past experience of violence by a household member. Such a household level approach may be of particular utility in settings of urban poverty in low to middle income countries, where mental health resources and services are severely constrained.

## Supporting Information

S1 Dataset (DTA)

S1 Table Risk factor model 2. (DOCX)
