# Supplementary material for: Household Factors Associated with Self-Harm in Johannesburg, South African Urban-Poor Households
Source: PLoS One. 2016 Jan 5;11(1):e0146239. doi: 10.1371/journal.pone.0146239 (PMC4701361; doi:10.1371/journal.pone.0146239)
Supplement: S1 Table — (DOCX) [file pone.0146239.s002.docx]

**Supplementary table 1a: Base model factors associated with self-harm: Johannesburg, South Africa (2006 - 2011).**

| **Risk Factor** | **Crude OR**  **(95% CI)** | ***p*-value** |
| --- | --- | --- |
| Household size | 1.89 (1.26-2.83) | **0.002** |
| Study site | 1.02 (0.82-1.26) | **0.853** |
| Household education | 1.26 (0.58-2.72) | **0.556** |
| Household employment | 1.39 (0.78-2.49) | **0.267** |
| Household income | 1.38 (0.84-2.22) | **0.188** |

**Supplementary table 1b: Significant risk factors associated with self-harm: Johannesburg, South Africa (2006 - 2011).**

| **Risk Factor** | **OR**  **(95% CI)** | ***p*-value** |
| --- | --- | --- |
| Household size | 1.72 (1.03- 2.89) | 0.038 |
| Study site | 0.82 (0.61-1.09) | 0.182 |
| Household education | 0.72 (0.26-1.97) | 0.519 |
| Household employment | 1.46 (0.61-3.52) | 0.398 |
| Household income | 1.52 (0.76-3.02) | 0.237 |
| Smoking inside the house | 2.41 (1.03-5.63) | **0.042*** |
| Household member victim of violent crime | 6.68 (2.88-15.48) | **0.000*** |
| Household member victim of non-violent crime | 1.18 (0.45-3.10) | 0.739 |
| Household member convicted of crime | 1.98 (0.71-5.49) | 0.188 |
| Household member suffers from chronic disease | 5.68 (2.71-11.86) | **0.000*** |
| Household member suffers from mental illness | 1.01 (0.22-4.67) | 0.994 |
| Death in household in last year | 1.94 (0.65-5.83) | 0.237 |

- Significant risk factors after base model factors included.
